# Factor structure of the patient health questionnaire-9 and measurement invariance across gender and age among Chinese university students

**DOI:** 10.1097/MD.0000000000032590

**Published:** 2023-01-06

**Authors:** Yang Wang, Lijuan Liang, Zhenyuan Sun, Rongxun Liu, Yange Wei, Shisan Qi, Qiao Ke, Fei Wang

**Affiliations:** a Psychology Institute, Inner Mongolia Normal University, Hohhot, Inner Mongolia, P.R. China; b Early Intervention Unit, Department of Psychiatry, Affiliated Nanjing Brain Hospital, Nanjing Medical University, Nanjing, P.R. China; c Functional Brain Imaging Institute, Nanjing Medical University, Nanjing, P.R. China; d Laboratory of Psychology, The First Affiliated Hospital of Hainan Medical University, Haikou, Hainan, P.R. China; e University of Toronto, Toronto, Ontario, Canada; f College of Medical Engineering, Xinxiang Medical University, Xinxiang, Henan, P.R. China; g Graduate School, Medical University Nanjing, Nanjing, P.R. China.

**Keywords:** Chinese university students, factor structure, gender and age, measurement invariance, patient health questionnaire

## Abstract

The Patient Health Questionnaire-9 (PHQ-9) has been widely used to screen depression symptoms. The present research aimed to assess the reliability and validity of PHQ-9, besides measurement invariance of the PHQ-9 across gender and age among Chinese university students. A total of 12,957 Chinese college students from 2 universities in Henan and Hainan provinces (China) completed the questionnaires via WeChat. This research reported the psychometric properties of PHQ-9 and measurement invariance of the PHQ-9 across gender and age among Chinese university students. Compared with 1-factor model, the 2-factor (affective factor and somatic factor) model of PHQ-9 showed a better fit index in Chinese university students. Without the last 2 items, the 2-factor model of the PHQ-9 showed satisfactory reliability, validity, and good fit index (e.g., Root mean square error of approximation = 0.060, Goodness-of-fit index = 0.982, Comparative fit index = 0.986, and Tucker-Lewis index = 0.974). The Cronbach’s alpha of PHQ-9 was 0.874. Multi-group analysis across gender and age demonstrated that measurement equivalency for the 2-factor model of the PHQ-9 was established (e.g., Root mean square error of approximation < 0.08, Comparative fit index > 0.90 and Tucker-Lewis index > 0.90). The 2-factor model of the PHQ-9 without the items of “movement” and “desire to die” showed a better fit index in Chinese university students. The measurement equivalence across gender and age for the 2-factor model of the PHQ-9 can be established among Chinese university students.

## 1. Introduction

The World Health Organization (WHO) defined depression as the fourth global leading cause of disability, accounting for 3.7% of all disability-adjusted life years.^[1]^ Depression is a consequence of complex interactions between biological, social and psychological factors. Studies have shown that in recent years, the incidence of depression has been increasing worldwide year by year.^[2–4]^ At present, several scoring systems have been utilized to measure depressive symptoms, while the majority of them include more than 10 items and may be inconsistent with the diagnostic criteria for depressive disorders.^[5,6]^ Therefore, other effective and brief scoring systems need to be developed to screen depression symptoms among university students.

The Patient Health Questionnaire (PHQ) is a new instrument for making criteria-based diagnoses of depression and other mental disorders commonly encountered in primary care. The patient health questionnaire-9 (PHQ-9) is the depression module, which scores each of the 9 DSM-IV (the Fourth Edition of Diagnostic and Statistical Manual of Mental Disorders) criteria as “0” (not at all) to “3” (nearly every day).^[7,8]^ A number of scholars have demonstrated that PHQ-9 can effectively screen depressive symptoms, accompanied by satisfactory sensitivity and specificity.^[9–11]^ PHQ-9 can assess somatic and non-somatic symptoms of depression, and even the risk of suicide. The PHQ-2, consisting of the first 2 items of the PHQ-9, is a well-studied screening measure of depression often used in primary care. There are 2 cardinal symptoms of major depression, sometimes referred to as “gateway” symptoms, because at least 1 must be present for the patient to meet the diagnostic criteria for the illness. These symptoms are depressed mood and diminished interest or pleasure in all, or nearly all, activities (anhedonia). These 2 cardinal symptoms are assessed by the PHQ-2.^[12]^

Several scholars applied PHQ-9 to screen depressive symptoms in patients with and without mental health problems.^[8,13]^ Meanwhile, PHQ-9 has been utilized to discriminate depression symptoms of the general population effectively,^[14]^ and it also has been employed to assess depression symptoms to explore the underlying psychological and behavioral mechanisms.^[3]^ Previous research demonstrated that PHQ-9 is of great significance in estimating depression symptoms amongst Ethiopian,^[15]^ Nigerian,^[14]^ Colombian,^[16]^ Korean,^[17]^ and Chinese^[18]^ university students. The Chinese version of the PHQ-9 was applied to screen depressive symptoms in the Chinese population, which showed satisfactory sensitivity and specificity.^[19,20]^ For university students, PHQ-9 was used to screen their depressive symptoms.^[13]^

Several scholars have shown that depression may associate with gender and age, while females and young adults have shown a stronger correlation with factors of gender and age, respectively.^[21]^ Evidence on the measurement invariance of PHQ-9 according to sex is less consistent than in other group comparisons. Studies supported a strong invariance for sex comparisons, whereas other studies reported weak or no measurement invariance across sexes, indicating that men and women interpret the PHQ-9 items differently.^[22]^

The PHQ-9 shows measurement invariance totally for women and partially for men in a study of a Dutch sample, particularly for the item “psychomotor problems.^[23]^ A number of researchers assessed the measurement invariance of the standard 1-factor model of the PHQ-9 and 5 previously identified 2-factor models for 4443 American Indian and 4443 Caucasian American adults (age ≥ 18 years old) with a PHQ-9. All models showed good fits (e.g., Comparative fit index (CFI) > 0.99, root mean square error of approximation (RMSEA) < 0.05) and internal consistency reliability (ordinal alpha > 0.80).^[24]^ All models displayed measurement invariance between racial groups. Factor correlation was high for 2-factor models, providing support for the 1-factor model. American Indian adults had significantly higher odds of PHQ-9 total scores ≥ 10 and ≥ 15 than Caucasian American adults.^[24]^ A recent study examined measurement invariance for the PHQ-9 by participants’ sex, recruitment stratum, and linguistic background. Results supported the invariance of PHQ-9 across sex, patient strata and linguistic background. The adequate psychometric properties for PHQ-9 allow direct multi-group comparison across sex, strata, and linguistic background.^[25]^ PHQ-9 has a 2-factor structure for persistent major depressive disorder patients, with solid measurement invariance between treatment groups at and across follow-up time.^[26]^ However, previous research also reported that PHQ-9 was not measurement invariant across Germans without a migration background and asylum seekers living in Germany; even with the same latent level of depression, asylum seekers may have higher scores on several items and consequently a higher sum score.^[27]^ The present research aims to assess the reliability and validity of PHQ-9. The factor structure and measurement invariance of the PHQ-9 across sex and age among Chinese university students are also performed.

## 2. Materials and Methods

### 2.1. Participants and procedure

A total of 12,957 Chinese university students from 1 university in Henan and Hainan provinces (China) completed the questionnaires via WeChat. Except for 99 university students who provided incomplete data anonymously, 12,858 students (4489 males and 8369 females) were included in the current survey. The age of the participants ranged from 17 to 25 years old (mean age, 21.17 ± 1.96).

All participants participated in this study voluntarily and signed the online informed consent before participation. Ethical approval for this study was obtained from Xinxiang Medical University Ethics Committee (HYLL2020005). All questionnaires were administered on the public account platform of WeChat. The psychology teacher instructed the students to finish the basic information and complete the questionnaire online. At the same time, all subjects were able to obtain timely feedback on their depression status after completing the measurement.

### 2.2. Measurement

PHQ-9 includes 9 items and is usually utilized to assess the severity of depressive symptoms by self-reporting. The 9 items were consistent with the DSM-IV criteria for major depressive disorder, and 2 subscales of somatic and non-somatic symptoms were involved. The related research indicated that PHQ-9 showed Cronbach’s of 0.89 among patients.^[8]^ Subjects rated the frequency of the 9 symptoms over the past 2 weeks on a 4-point Likert scale (0 = not at all, 1 = several days, 2 = more than half of the days, and 3 = nearly every day). The total score of the PHQ-9 ranged from 0 to 27, and higher scores indicated greater severity of depressive symptoms.^[8]^ According to the Chinese version of the PHQ-9, 0-4 is normal, 5 to 9 is mild, 10-14 is moderate, 15 to 19 is moderate severe, and 20 to 27 is severe depression symptoms.^[28]^

### 2.3. Data analysis

The data were analyzed using SPSS Statistics version 23 (IBM, Armonk, NY) and AMOS version 21. Results were presented as mean ± standard deviation. According to the symptom scores of PHQ-9, the basic depression status of university students was analyzed. For further analysis, the distribution of the data was analyzed as well.

In order to explore the factor structure, our analysis included 2 steps. Firstly, exploratory factor analysis (EFA) was conducted on a randomly split-half of the whole sample (n = 6386) to identify the best fitting factor model of the PHQ-7 in university students. Secondly, Confirmatory factor analysis (CFA) was performed using the remaining sample (n = 6472). Model fit was assessed with various fit indices, including Chi-square with estimated degree of freedom, CFI, Tucker-Lewis index (TLI), and RMSEA. RMSEA < 0.08 and CFI and TLI > than 0.90 indicated acceptable models.^[29]^

Based on the good factor structure, the reliability of the PHQ-9 was estimated with Cronbach’s alpha. In the case of Cronbach’s alpha > 0.5,^[30]^ construct reliability (CR) and average variance extracted (AVE) were calculated. After the identification of the best fitting model, measurement invariance tests were conducted across gender and age to assess the effectiveness of inter-group comparisons. Five aspects of invariance were considered, including the unconstrained model, measurement weights model, measurement intercepts model, structural covariances model and measurement residuals model. First, the unconstrained model is the basic model of equivalence, which refers to the pattern equivalence of the model. Based on pattern invariance, the factor loading invariance and measurement intercept of different groups are limited to be equivalent. Finally, based on the above 3 models, this study further explored whether the covariance and residuals are the same across different groups. The CFI, TLI and RMSEA were used to evaluate invariance across consecutive models; besides, in the case of CFI and TLI ≥ 0.9, measurement invariance was accepted, and CFI, TLI, and RMSEA changes were employed to evaluate invariance; ΔCFI ≤ 0.01, ΔTLL ≤ 0.01, and ΔRMSEA ≤ 0.015 were considered evidence of invariance.^[29]^

## 3. Results

### 3.1. Descriptive statistics

In the whole sample, the mean PHQ-9 score was 3.26 ± 4.12 for males and 3.49 ± 3.90 for females. Gender-based differences were statistically significant (*t* = 3.082, *P* = .002) (Table 1). All the participants were assigned to 3 age-based subgroups: 17 to 19 years old (n = 3027), 20 to 22 years old (n = 6631), and 23 to 25 years old (n = 3636). Meanwhile, the mean PHQ-9 scores for the groups of 17 to 19, 20 to 22, and 23 to 25 years old were 3.41 ± 3.86, 3.50 ± 4.03, and 3.24 ± 3.97, respectively. Age-based differences were statistically significant (*F* = 4.838, *P* = .008). According to the cutoff value of the PHQ-9, 30.2% of the participants had depression (PHQ-9 score ≥ 5), including 8971 (69.8%) with normal, 2967 (23.1) with mild, 630 (4.9%) with moderate, 201 (1.6%) with moderately severe, and 89 (0.7%) with severe depression symptoms.^[8]^ The majority of the items were normally distributed, except for the last 2 items, “movement” and “desire to die.” There were 85 (0.7%) and 31 (0.3%) students with severe “movement” and “desire to die,” respectively.

**Table 1:** Baseline demographic of the total sample.

	Frequency	Percent	Mean	SD	*t*/*F*	*p*
Gender					3.082	.002
Male	4489.000	34.900	3.260	4.120		
Female	8369.000	65.100	3.490	3.900		
Age					4.838	.008
17–19	3008.000	23.394	3.410	3.860		
20–22	6552.000	50.957	3.500	4.030		
23–25	3298.000	25.649	3.240	3.970		
Depression symptom					22,790.013	.000
Normal	8971.000	69.770	1.302	1.413		
Mild	2967.000	23.075	6.620	1.354		
moderate	630.000	4.900	11.479	1.334		
Moderately severe	201.000	1.563	16.632	1.383		
Severe	89.000	0.692	22.416	2.022		
History of smoking					6.864	.000
No	12,350.000	96.049	3.346	3.890		
Yes	508.000	3.951	5.055	5.557		
History of drinking					11.252	.000
No	11,031.000	85.791	3.220	3.785		
Yes	1827.000	14.209	4.560	4.858		
History of Psychiatry					7.932	.000
No	12,789.000	99.463	3.381	3.943		
Yes	69.000	0.537	9.362	6.257		

#### 3.1..1. EFA.

Since the factor structure of PHQ-9 was controversial, EFA was utilized to determine the applicability of the 2- or 1-factor structure of PHQ-9.^[13]^ Factor analyses with oblique rotation yielded a KMO value of 0.927 for the PHQ-9, and the Bartlett test of sphericity indicated a *χ*2 value of 25,153.214 (*P* < .001). Based on the recent research^[31]^ and the large sample sizes in our study, the factor extraction criteria is 0.5. Except for items of “movement” and “desire to die,” all factor loading of all items were more than 0.5. The standard 2-factor accounted cumulatively for 52.64% of the total variance. EFA factors and factor loadings are presented in Table 2. The skewness for a normal distribution is less than 1, and any symmetric data should have a skewness near zero; the kurtosis for a standard normal distribution is less than 10.^[32]^ The normality test showed that the skewness of all items were less than 3, and the kurtosis of all items were less than 10, except item 8 and 9, which demonstrated all other 7 items conformed to the normal distribution and were suitable for future data analysis. Followed-up analysis excepted the last 2 items due to the low frequency and unnormal distribution.

**Table 2:** Factor loadings for EFA in sample 1.

Item content	Depression	Mean (SD)	Skewness	Kurtosis
1. Little interested or pleasure in doing things	0.805	0.59 (0.72)	1.258	1.633
2. Feeling down, depressed, or hopeless	0.787	0.44 (0.60)	1.315	2.027
3. Trouble falling or staying asleep, or sleeping too much	0.740	0.49 (0.75)	1.635	2.392
4. Feeling tired or having little energy	0.834	0.54 (0.70)	1.321	1.772
5. Poor appetite or overeating	0.737	0.36 (0.65)	2.042	4.267
6. Feeling bad about yourself – or that you are a failure or have let yourself or your family down	0.753	0.39 (0.65)	1.796	3.431
7. Trouble concentrating on things, such as reading the newspaper or watching television	0.701	0.39 (0.67)	1.857	3.398
Movement	0.667	0.18 (0.06)	3.230	4.730
Desire to die	0.547	0.08 (0.06)	12.07	27.39
Explained variance	52.64%			

EFA = exploratory factor analysis.

#### 3.1..2. CFA.

**Figure 1.**
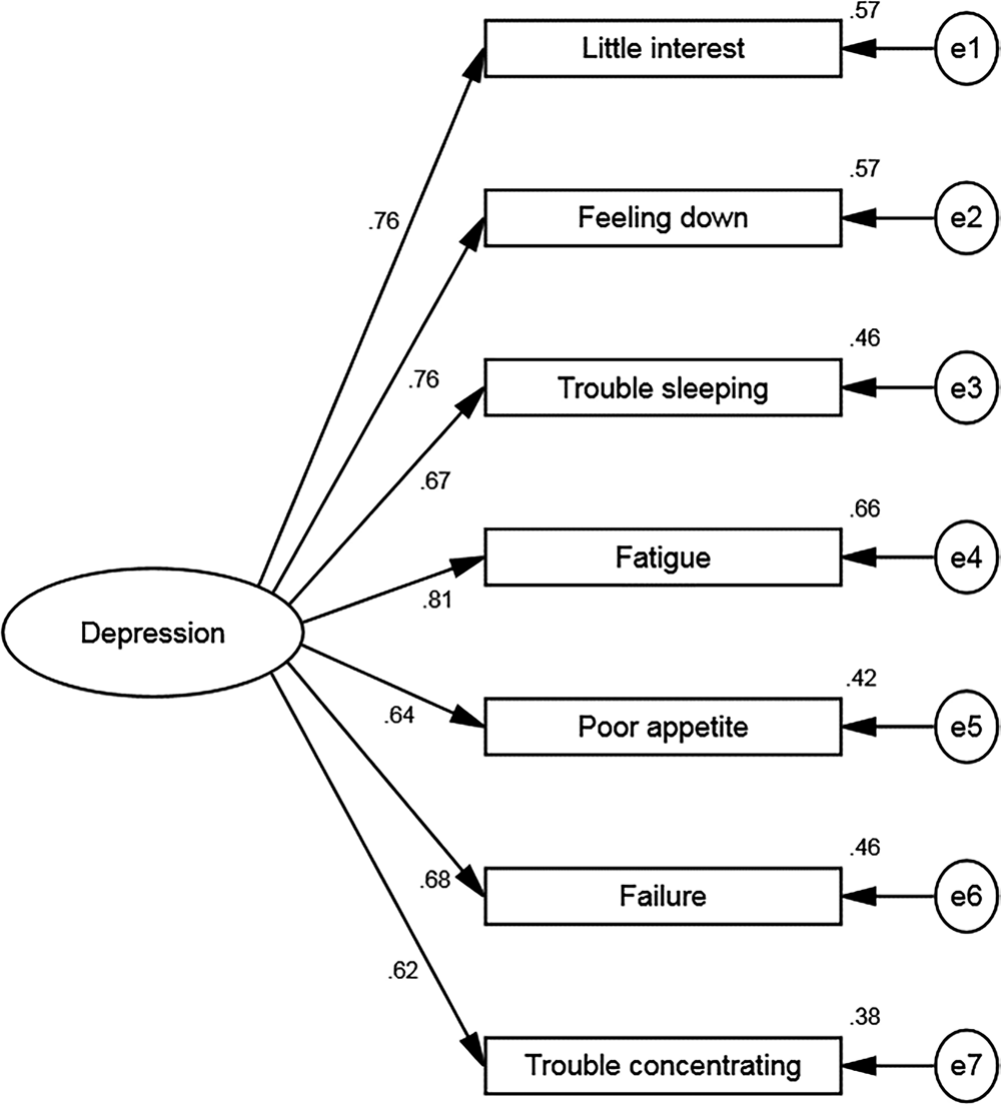
The standardized factor loadings of the models.

The standardized factor loadings of the models are displayed in Figure 1. The factor loadings of 7 items of the PHQ-9 ranged from 0.62 to 0.82 (Fig. 1). Compared with the 1-factor model of PHQ-9, the 2-factor model showed a more satisfying model fit index. The CFA revealed adequate-to-excellent fit to the data for standard 2-factor model of the PHQ-9 (*χ*2 (13) = 325.729, *P* = .000, CFI = 0.984, Goodness-of-fit index = 0.986, TLI = 0.974, and RMSEA = 0.060 (90% CI: [0.054,0.066] 0.054,0.066) (Table 3).

**Table 3:** Model fit index for competitive models.

Model	c^2^ (*df*)	c^2^/*df*	RMSEA	LO 90	HI 90	CFI	TLI	GFI
One-factor of PHQ-7	424.893 (14)^a^	30.350	0.066	0.061	0.072	0.979	0.968	0.982
Two-factor of PHQ-7	325.729 (13)^a^	25.056	0.060	0.054	0.066	0.984	0.974	0.986

Note: *df* = degree of freedom, TLI = Tucker-Lewis index, CFI = comparative fit index, RMSEA = root mean square error of approximation, LO90 = lower boundary of a two-sided 90% confidence interval, HI90 = upper boundary of a two-sided 90% confidence interval.

a *p *< 0.001.

### 3.2. Reliability and evidence of validity

The Cronbach’s alpha of PHQ-9 was 0.874, which was greater than 0.6.^[30]^ Pearson’s correlation analysis was undertaken to examine the reliability of PHQ-9. The total scores and 7 items were also significantly and strongly correlated. To further verify the reliability and validity of the PHQ-9, CR and AVE were calculated, and the results showed that the CRs of 2 factors were 0.788 and 0.791, respectively, and the AVEs of 2 factors were 0.554 and 0.489. Thus, the reliability and validity of PHQ-9 were confirmed.^[33]^

### 3.3. Factorial invariance across gender

Multiple-group analysis was carried out to examine the gender-based structural equivalence of the 2-factor model of the PHQ-9. The unconstrained model fit indices were obtained TLI = 0.961, CFI = 0.974, RMSEA (90% CI) = 0.052 (0.48, 0.056). Each fit index met the requirements of configural invariance for the 1-factor model of the PHQ-9 (Table 4).

**Table 4:** Goodness-of-fit indices of one-factor PHQ-7 for measurement invariance models across gender.

Model	c^2^ (*df*)	c^2^/*df*	RMSEA	LO90	HI90	CFI	TLI	∆CFI	∆TLI
Unconstrained Model	524.302 (28)^a^	18.725	0.052	0.048	0.056	0.974	0.961		
Measurement Weights Model	541.629 (34)^a^	15.930	0.048	0.045	0.052	0.973	0.967	0.001	0.006
Measurement Intercepts Model	562.12 (41)^a^	13.710	0.044	0.041	0.048	0.973	0.972	0.000	0.005
Structural Covariances Model	565.455 (42)^a^	13.463	0.044	0.041	0.047	0.973	0.973	0.000	0.001
Measurement Residuals Model	600.807 (49)^a^	12.261	0.042	0.039	0.045	0.971	0.975	0.002	0.002

Note: *df* = degree of freedom, TLI = Tucker-Lewis index, CFI = comparative fit index, RMSEA = root mean square error of approximation, LO90 = lower boundary of a two-sided 90% confidence interval, HI90 = upper boundary of a two-sided 90% confidence interval.

a *p *< .001.

According to the measurement equivalence, ΔCFI and ΔTLI of the second model were 0.001 and 0.006, respectively.^[29]^ The measurement intercept of the 1-factor model of the PHQ-9 showed that intercept equivalence was established, as both ΔCFI and ΔTLI were less than 0.01. Based on a previous model, we tested the structural covariance of the 1-factor model of the PHQ-9. The fit indices of the structural covariance model revealed that ΔCFI and ΔTLI were equal to 0.000 and 0.001, respectively. Accordingly, the fit index of measurement residual invariance showed good fit indices (TLI = 0.975, CFI = 0.971, RMSEA = 0.042), and ΔCFI and ΔTLI were equal to 0.002 (Table 4).

### 3.4. Factorial invariance across different age-based groups

Multi-group analysis was conducted to examine the age equivalence of the model of 1 factor PHQ-9. Except for significant differences achieved from Chi-square, all other fit indices of models were good, as summarized in Table 5. The RMSEA of all examined models was less than 0.05, while the CFI and ΔCFI of the 5 examined models were more than 0.9 and 0.01, respectively. In addition, TLI and ΔTLI were also more than 0.9 and less than 0.01, respectively, indicating a good fit (Table 5).

**Table 5:** Goodness-of-fit indices of one-factor PHQ-7 for measurement invariance models across age.

Model	c^2^ (*df*)	c^2^/*df*	RMSEA	LO90	HI90	CFI	TLI	∆CFI	∆TLI
Unconstrained Model	645.608 (63)^a^	10.248	0.038	0.035	0.040	0.969	0.969		
Measurement Weights Model	655.511 (69)^a^	9.500	0.036	0.034	0.039	0.969	0.972	0.000	0.003
Measurement Intercepts Model	660.607 (76)^a^	8.692	0.034	0.032	0.037	0.969	0.975	0.000	0.003
Structural Covariances Model	660.836 (77)^a^	8.582	0.034	0.032	0.037	0.969	0.975	0.000	0.000
Measurement Residuals Model	686.187 (84)^a^	8.169	0.033	0.031	0.036	0.968	0.976	0.001	0.001

Note: *df* = degree of freedom, TLI = Tucker-Lewis index, CFI = comparative fit index, RMSEA = root mean square error of approximation, LO90 = lower boundary of a two-sided 90% confidence interval, HI90 = upper boundary of a two-sided 90% confidence interval.

a *p *< .001.

## 4. Discussion

The present research showed that several university students suffered from depressive symptoms, while most reported mild or moderate depressive symptoms. Compared with male university students, female university students experienced more severe depressive symptoms. Meanwhile, compared with other age-based groups, participants in the group middle-age group (20–22 years old) had significantly more depressive symptoms. Compared with younger university students, older university students experienced a higher pressure of study and employment. The management of mental health disorders among university students may be realized by alleviating university students’ psychological problems. However, due to academic stress,^[34]^ interpersonal relationships,^[35]^ and the importance of employment,^[36]^ further attention should be paid to university students, especially female students and senior students. Since most Chinese university students did not report severe psychiatric symptoms, such as “movement” and “desire to die,” the frequency of the 2 items was low, so these 2 items were non-normally distributed.

The PHQ-9 is mainly utilized to evaluate depressive symptoms, while it may include somatic and non-somatic symptoms.^[16]^ The factor structure of PHQ-9 has remained a controversial topic. EFA results showed that the 2-factor structure of PHQ-9 is more appropriate for university students rather than the 1-factor structure. This result is also consistent with the finding of previous research.^[8]^

The results of CFA revealed that the structural validity of the PHQ-9 was acceptable, and the items of the PHQ-9 could effectively represent latent variables. Compared with the clinical psychiatric sample, the factor loading of the university sample was relatively lower as most university students did not report severe depressive symptoms.^[37]^ Compared with other symptoms, the first 2 items of the PHQ-9 also showed higher factor loading, which was consistent with outcomes of previous research.^[12,19]^ However, many university students reported mild or moderate depression symptoms, and a low proportion of university students had reported movement and suicidal thoughts.

Without reaching acceptable reliability and validity, using the PHQ-9 may result in under-detection or over-detection of depression symptoms in certain groups.^[38]^ The Cronbach’s alpha in the PHQ-9 was consistent with previously reported research that presented outstanding reliability in mental health patients, non-mental health patients,^[14,39]^ and the general population.^[18,20,40]^ According to the current survey results, the 2-factor model of the PHQ-9 is highly recommended for university students.

For the superior reliability and validity of PHQ-9 without the last 2 items, we only analyzed the measurement equivalency of PHQ-9 in the current research. Because of the large sample size of this research, chi-square values were easily affected by the sample size, which may lead to significant differences. Although relevant research also explored the equivalence of PHQ-9 among middle school students of Hong Kong,^[41]^ the equivalence of PHQ-9 was established across age and gender. However, the reliability and validity were not reported in that research. With different age groups and different cultural backgrounds, the scale may only be partially applicable. Therefore, this study further explored the reliability, validity and equivalence of PHQ-9 in Chinese university students. Similar to younger Chinese adolescents, measurement invariances of the PHQ-9 across gender- and age-based subgroups in Chinese university students were also confirmed. In this research, the 2-factor model of the PHQ-9 met the requirements for configural equivalence. Therefore, the next step of the equivalence test could be carried out. The establishment of the measurement weight invariance indicated that the observed and potential traits of the PHQ-9 have the same meaning in different gender- and age-based groups. Therefore, the latent variables of the PHQ-9 and the meaning of the observed items are equivalent in different groups. Hence, the intercept of indicators and structural co-variances were further analyzed. The results of the current survey revealed that the intercept of indicators and structural co-variances were satisfied. This finding also was consistent with previously reported outcomes.^[42]^

There are some limitations in the present research. This research collected data from 3 medical universities in 2 provinces of China, while there might be some differences in depression symptoms of students from different universities. Therefore, in the next study, the level of education and type of school should be considered. Additionally, self-report assessment may also have some shortcomings. Therefore, other methods investigating depression symptoms can be used in combination to reach higher clinical efficacy. In addition, retest reliability may be beneficial to explore the stability, and other validity, such as convergent or discriminating validity, should be considered to further conduct for better certification of the measurement properties.

## 5. Conclusion

Compared with 1-factor model, the 2-factor (affective factor and somatic factor) model of PHQ-9 without the items of “movement” and “desire to die” showed a better fit index in Chinese university students. The 2-factor structure model was found highly appropriate for university students and indicated satisfactory reliability and validity. The measurement equivalence across different genders and ages was established using the 2-factor model of the PHQ-9 for Chinese university students among Chinese university students.

## Acknowledgements

This study was funded by National Science Fund for Distinguished Young Scholars (81725005 to Fei Wang), National Natural Science Foundation Regional Innovation and Development Joint Fund (U20A6005 to Fei Wang), Jiangsu Provincial Key Research and Development Program (BE2021617 to Fei Wang), Inner Mongolia Autonomous Region Postgraduate Education Innovation Program Funding Project (B202101194Z to Yang Wang), Hainan Provincial Natural Science Foundation of China (821RC700 to Lijuan Liang), Henan Province Higher Education Teaching Reform Research and Practice Project (2021SJGLX189 to Yongxun Liu).

## Author contributions

**Conceptualization:** Lijuan Liang, Shisan Qi, Fei Wang.

**Data curation:** Yang Wang.

**Formal analysis:** Yang Wang.

**Funding acquisition:** Yang Wang, Rongxun Liu, Shisan Qi, Fei Wang.

**Investigation:** Lijuan Liang, Rongxun Liu, Yange Wei, Fei Wang.

**Methodology:** Lijuan Liang, Qiao Ke.

**Project administration:** Qiao Ke.

**Resources:** Yange Wei.

**Software:** Yang Wang, Yange Wei.

**Supervision:** Yang Wang, Zhenyuan Sun, Qiao Ke.

**Validation:** Lijuan Liang, Rongxun Liu.

**Visualization:** Lijuan Liang.

**Writing – original draft:** Lijuan Liang.

**Writing – review & editing:** Yang Wang, Zhenyuan Sun, Yange Wei, Fei Wang.
